# *Ehrlichia chaffeensis* and *E. canis* hypothetical protein immunoanalysis reveals small secreted immunodominant proteins and conformation-dependent antibody epitopes

**DOI:** 10.1038/s41541-020-00231-1

**Published:** 2020-09-11

**Authors:** Tian Luo, Jignesh G. Patel, Xiaofeng Zhang, David H. Walker, Jere W. McBride

**Affiliations:** 1 Department of Pathology, University of Texas Medical Branch, Galveston, TX USA; 2 Center for Biodefense and Emerging Infectious Diseases, University of Texas Medical Branch, Galveston, TX USA; 3 Sealy Institute for Vaccine Sciences, University of Texas Medical Branch, Galveston, TX USA; 4 Institute for Human Infections and Immunity, University of Texas Medical Branch, Galveston, TX USA; 5 Department of Microbiology and Immunology, University of Texas Medical Branch, Galveston, TX USA

**Keywords:** Infectious diseases, Vaccines, Bacteria, Infectious-disease diagnostics, Vaccines

## Abstract

Immunomolecular characterization of *Ehrlichia chaffeensis* (*E. ch*.) and *E. canis* (*E. ca*.) has defined protein orthologs, including tandem repeat proteins (TRPs) that have immunodominant linear antibody epitopes. In this study, we combined bioinformatic analysis and cell-free protein expression to identify undiscovered immunoreactive *E. ch*. and *E. ca*. hypothetical proteins. Antigenicity of the *E. ch*. and *E. ca*. ORFeomes (*n* = 1105 and *n* = 925, respectively) was analyzed by the sequence-based prediction model ANTIGENpro, and we identified ~250 ORFs in each respective ORFeome as highly antigenic. The hypothetical proteins (*E. ch*. *n* = 93 and *E. ca*. *n* = 98) present in the top 250 antigenic ORFs were further investigated in this study. By ELISA, 46 *E. ch*. and 30 *E. ca*. IVTT-expressed hypothetical proteins reacted with antibodies in sera from naturally *E. ch*.-infected patients or *E. ca*.-infected dogs. Moreover, 15 *E. ch*. and 16 *E. ca*. proteins consistently reacted with a panel of sera from patients or dogs, including many that revealed the immunoreactivity of “gold standard” TRPs. Antibody epitopes in most (>70%) of these proteins exhibited partial or complete conformation-dependence. The majority (23/31; 74%) of the major immunoreactive proteins identified were small (≤250 aa), and 20/31 (65%) were predicted to be secreted effectors. Unlike the strong linear antibody epitopes previously identified in TRP and OMP orthologs, there were contrasting differences in the *E. ch*. and *E. ca*. antigenic repertoires, epitopes and ortholog immunoreactivity. This study reveals numerous previously undefined immunodominant and subdominant antigens, and illustrates the breadth, complexity, and diversity of immunoreactive proteins/epitopes in *Ehrlichia*.

## Introduction

*Ehrlichia chaffeensis* (*E. ch*.) and *E. canis* (*E. ca*.) are tick-transmitted obligately intracellular bacteria that cause human monocytotropic ehrlichiosis (HME) and canine monocytic ehrlichiosis (CME), respectively^1^. HME is an emerging life-threatening zoonosis in humans, with 50–70% of the cases requiring hospitalization and a fatality rate of ~3%^2^. CME is a globally distributed disease in dogs and is the most serious form of canine ehrlichiosis^3^. Currently therapeutic options are limited and there are no vaccines available for HME or CME. Progress in developing effective subunit vaccines for HME and CME has been hindered by many factors, not the least of which is the small and incomplete repertoire of molecularly defined *E. ch*. and *E. ca*. protective proteins^4^.

Previous studies have identified a small group of major immunoreactive protein orthologs of *E. ch*. and *E. ca*., including the tandem repeat proteins (TRPs)^5–9^, an ankyrin repeat protein (Ank200)^10,11^, and the major outer membrane protein (OMP) family^12,13^ with linear antibody epitopes that have been molecularly defined. During infection, strong TRP-specific, Ank-specific, and OMP-specific antibody responses are consistently generated in humans and dogs that can be demonstrated by immunoblot^14,15^. Moreover, linear antibody epitopes of *E. ch*. TRPs and OMP-1 have been shown to stimulate antibodies that are protective^16,17^. Although protective linear epitopes in *Ehrlichia* spp. have been defined, there are limited examples of conformation-dependent antibody epitopes, and there is little knowledge regarding the existence of such epitopes or their roles in immunity.

The established antigenic repertoire of *E. ch*. and *E. ca*. consists of ~10 immunodominant proteins known to be expressed in *Ehrlichia-*infected mammalian cells^4^. These proteins were identified using approaches that depend on linear antibody epitopes; however, it is recognized that identification of the complete repertoire of antigenic proteins can be limited by immunoscreening approaches and other factors such as the host cell environment, which influences pathogen antigen expression^18–20^. Whole-proteome screening of other pathogens has revealed a surprisingly large number of immunoreactive proteins not previously identified with conventional approaches^21–23^. Proteome microarray identified 185 immunodominant proteins in obligately intracellular chlamydiae, and a large proportion (*n* = 75) of these proteins are hypothetical^24^. Similarly, studies with other pathogens have demonstrated that 7–20% of the proteins in a pathogen are immunoreactive^21–26^.

Several bioinformatic tools to predict protein antigenicity have been developed including ANTIGENpro, which has been validated using antibody-reactivity data obtained by protein microarray and reported protective proteins^27–29^. The accuracy of ANTIGENpro has been shown to be high (82%) in predicting known protective antigens, outperforming other antigenicity predictors^29^. ANTIGENpro has been successfully used to identify antigenic proteins in many pathogens including *Salmonella*, *Leishmania*, and *Corynebacterium* spp.^29,30^. In addition, in vitro transcription and translation (IVTT) is a useful high-throughput approach to screen a variety of proteins for functional studies, since this system can rapidly produce soluble protein in native conformation and overcome difficulties often encountered with protein expression in cell-based systems^31,32^.

In this study, we have applied ANTIGENpro antigenicity prediction and high-throughput expression and immunoscreening approaches to rapidly identify *E. ch*. and *E. ca*. immunoreactive proteins. Using this strategy, we have identified a group of undiscovered immunoreactive *E. ch*. and *E. ca*. hypothetical proteins, many with conformation-dependent epitopes. These findings represent a significant advance in defining the *E. ch*. and *E. ca*. immunome by revealing numerous previously undefined immunodominant and subdominant proteins and illustrating the breadth, complexity, and diversity of immunoreactive proteins/epitopes in *Ehrlichia* spp.

## Results

### Predicting *E. ch*. and *E. ca*. protein antigenicity with ANTIGENpro

Antigenicity of 1105 *E. ch*. and 925 *E. ca*. open-reading frames (ORFs; excluding RNA genes and pseudogenes) were predicted by ANTIGENpro. The antigenicity score of *E. ch*. and *E. ca*. proteins ranged from 0.01 to 0.97, with the top 250 proteins in the respective genomes scoring above a minimum threshold (~0.7) that has been shown to provide a balance of sensitivity, specificity, and accuracy in predicting protein antigenicity^29^. Well-characterized major immunoreactive proteins of *E. ch*. and *E. ca*., including TRPs, Ank200, OMP-1/OMP-A (P28/P30), and MSP4 family members, were represented in the ANTIGENpro top 250, indicating concordance between previous experimental data and ANTIGENpro prediction (Table 1). Among the top 250 *E. ch*. and *E. ca*. proteins, 93 (37%) and 98 (39%) proteins were annotated as hypothetical without any putative function assigned in IMG database. The TRPs and Ank200, previously annotated as hypothetical, were excluded from this group. Based on existing empirical TRP/Ank data and other studies suggesting hypothetical proteins are frequent targets of the host immune response, we focused on this group in this investigation. The *E. ch*. and *E. ca*. hypothetical proteins in the top 250 were ranked according to the ANTIGENpro score (from high to low) (Supplementary Tables 1 and 2).

**Table 1:** ANTIGENpro antigenicity score and overall rank of known *E. ch*. and *E. ca*. major immunoreactive proteins.

*Ehrlichia*	Protein	Tag no.	Rank	Antigenicity score
*E. ch*.	TRP47	0166	30	0.908
	TRP32	0170	49	0.881
	TRP120	0039	100	0.838
	TRP75	0558	130	0.802
	Ank200	0684	184	0.759
	OMP-1/P28 family	Multiple^a^	68–233	0.861–0.711
*E. ca*.	TRP36	0109	8	0.943
	TRP19	0113	43	0.906
	OMP-A/P30 family	Multiple^b^	49–236	0.902–0.714
	TRP140	0017	227	0.724
	Ank200	0365	237	0.714

^a^Tag numbers include 1121, 1125, 1126, 1127, 1130, 1131, 1133, 1134, 1137, 1140, 1142, and 1144.

^b^Tag numbers include 0563, 0831, 0833, 0896, 0905, 0906, 0911, 0913, 0914, 0915, 0916, 0917, and 0918.

### Expression, immunoscreening and identification of *E. ch*. and *E. ca*. hypothetical proteins

An IVTT system was used to express the *E. ch*. and *E. ca*. hypothetical proteins. To confirm IVTT expression, dot blots were performed using anti-His-tag antibody on randomly selected proteins (17 from *E. ch*. and 18 from *E. ca*.). The expression of all proteins was detectable and the negative control (IVTT reaction without plasmid) was not detectable (Supplementary Fig. 1).

**Fig. 1:**
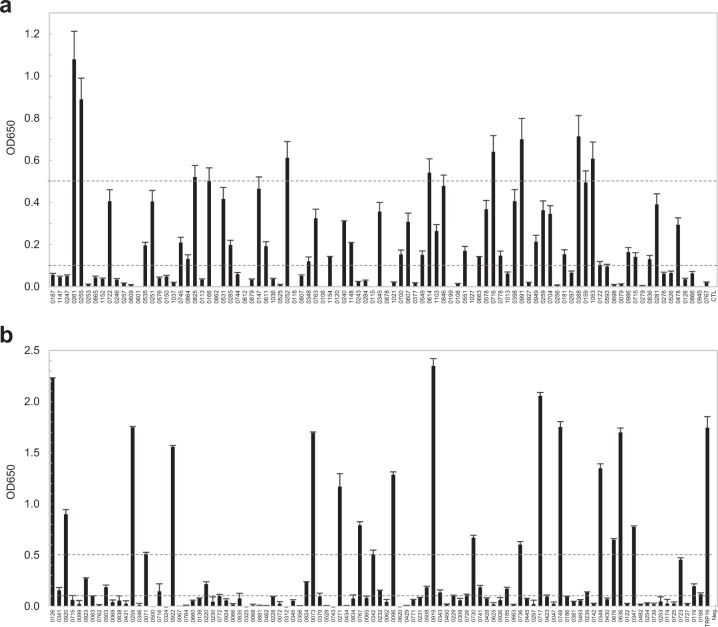
Immunoreactivity screening of *E. ch*. and *E. ca*. hypothetical proteins by ELISA. **a** An HME patient serum was used to screen *E. ch*. hypothetical proteins. **b** Pooled CME dog sera were used to screen *E. ca*. hypothetical proteins. ELISA OD values represent the mean optical density reading from three wells (±standard deviations) after background subtraction. A sample OD of ≥0.1 was considered positive and ≥0.5 a strong positive after subtracting negative control (IVTT reaction with the empty vector template) reading.

The 93 IVTT-expressed *E. ch*. proteins were screened for immunoreactivity by antigen capture ELISA using a single convalescent HME patient serum (IFA titer: 1600). A total of 46 (49%) *E. ch*. hypothetical proteins reacted with the HME patient serum (mean OD ≥ 0.1 with background subtracted) (Fig. 1a). The 98 IVTT-expressed *E. ca*. proteins were similarly screened for immunoreactivity by ELISA with pooled CME dog sera (IFA titer: 1600), and 38 (39%) proteins were immunoreactive (mean OD ≥ 0.1) (Fig. 1b). These immunoreactive *E. ch*. and *E. ca*. proteins were investigated further to determine overall immunoreactivity among a panel (*n* = 10) of HME and CME sera, respectively.

### Identification of major immunoreactive *E. ch*. and *E. ca*. hypothetical proteins

**Fig. 2:**
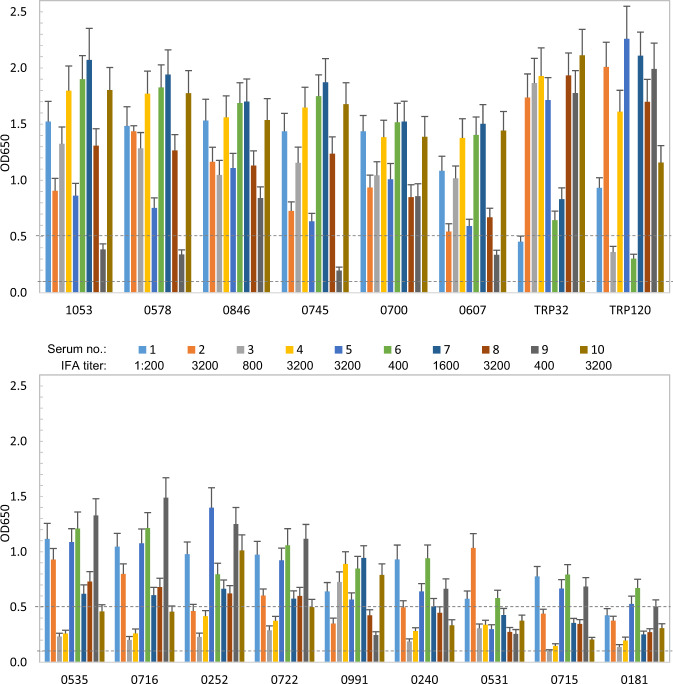
Immunoreactivity of 15 *E. ch*. hypothetical proteins and TRP comparison by ELISA. IVTT-expressed proteins were probed with a panel of convalescent sera from 10 patients diagnosed with HME. A normal human serum control did not recognize these proteins. ELISA OD values represent the mean optical density reading from three wells (±standard deviations) after background subtraction. A sample OD of ≥0.1 was considered positive and ≥0.5 a strong positive after subtracting negative control (IVTT reaction with empty plasmid template) reading. The top six proteins with mean OD of **≥**1.0 were considered immunodominant.

**Fig. 3:**
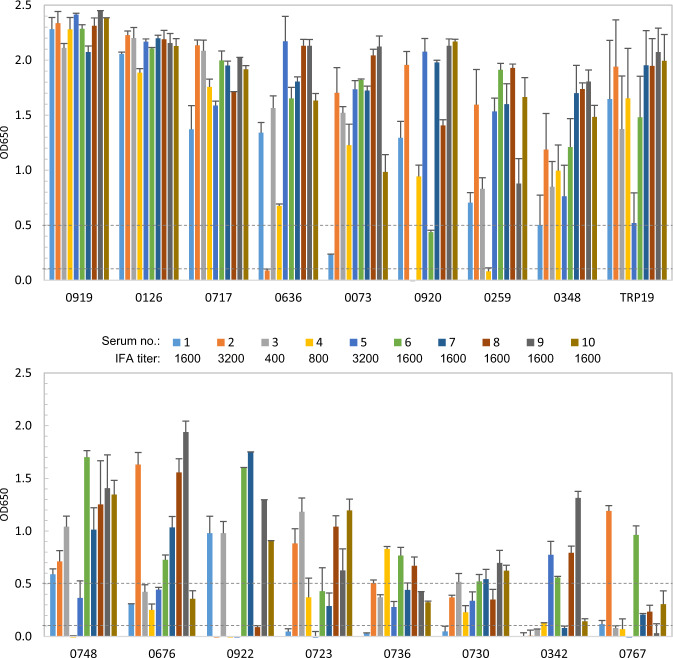
Immunoreactivity of 16 *E. ca*. hypothetical proteins and TRP19 comparison by ELISA. IVTT-expressed proteins were probed with convalescent sera from 10 dogs with CME. OD values represent the mean optical density reading from three wells (±standard deviations) after background subtraction. A sample OD of ≥0.1 was considered positive and ≥0.5 a strong positive after subtracting negative control (IVTT reaction with empty plasmid template) reading. The top eight proteins with mean OD of ≥1.0 were considered immunodominant.

In order to define and compare the immunoreactivity of these immunoreactive *E. ch*. and *E. ca*. hypothetical proteins, an ELISA was performed with a panel of 10 HME patient or 10 CME dog sera that had detectable *E. ch*. or *E. ca*. antibodies by IFA (titers ranging from 200 to 3200). To compare the immunoreactivity of newly identified *E. ch*. and *E. ca*. immunoreactive proteins with well-defined major immunoreactive TRPs, we also cloned and expressed *E. ch*. TRP32, TRP120, and *E. ca*. TRP19 by IVTT, and compared immunoreactivity of these proteins with HME and CME sera. Consistent with our previous data, *E. ch*. TRP32, TRP120 and *E. ca*. TRP19 reacted with all HME or CME sera (Figs 2 and 3).

Among 46 *E. ch*. immunoreactive proteins identified, 15 (33%) proteins were recognized by all HME patient sera (Fig. 2). These proteins ranked by mean ELISA OD values are shown in Table 2. The top six proteins reacted strongly with most HME patient sera, which in comparison with known immunodominant proteins (TRPs) were considered immunodominant based on mean ELISA OD values (≥1.0). In addition, six proteins reacted strongly with the majority of HME patient sera (mean OD 0.5–0.8), and three proteins reacted consistently, but at lower levels with HME patient sera (mean OD 0.3–0.5). Thus, these nine immunoreactive proteins were considered to be subdominant (Fig. 2 and Table 2). None of the HME patient sera reacted with the IVTT-expressed negative control protein (raw OD < 0.08). Eight (53%) of these immunoreactive proteins were ranked in the top 100 by ANTIGENpro, indicating the substantial enrichment of antigenic proteins in the top 100 tier.

**Table 2:** Immunodominant *E. ch*. hypothetical protein immunoreactivity and ANTIGENpro analysis.

Protein (Ech_ tag no.)	Mean ELISA OD^a^	ANTIGENpro rank	Antigenicity score	*E. ca*. ortholog/ANTIGENpro rank/Immunoreactive^b^
1053	1.39	180	0.762	0846/381/−
0578	1.39	137	0.797	*
0846	1.33	107	0.828	0242/417/−
0745	1.23	23	0.919	0324/26/−
0700	1.19	90	0.845	*
0607	1.00	92	0.811	0434/70/−
0535	0.80	18	0.927	0500/24/−
0716	0.78	142	0.790	0347/190/+
0252	0.78	55	0.874	*
0722	0.70	10	0.944	0342/80/+
0991	0.64	157	0.779	0139/32/−
0240	0.54	73	0.856	*
0531	0.45	33	0.904	*
0715	0.45	194	0.747	0348/178/+
0181	0.37	170	0.769	0122/189/−

^a^Mean OD from 10 HME patient sera.

^b^(+) Immunoreactive in immunoscreening; (−) not immunoreactive in immunoscreening; (*) *E.ca.* ortholog not identified.

Among 38 *E. ca*. immunoreactive proteins, we found that 16 (42% of 38) were recognized by most CME dog sera (Fig. 3). These proteins ranked by mean ELISA OD values are shown in Table 3. Top eight *E. ca*. proteins reacted strongly with most dog sera at a level comparable to TRP19 (mean OD > 1.0), thus were considered immunodominant. Another eight *E. ca*. proteins reacted with most dog sera but had mean ELISA OD values <1.0 and were classified as subdominant (Fig. 3 and Table 3). None of the CME patient sera reacted with the IVTT-expressed negative control protein (raw OD < 0.08). Seven (44%) of these proteins were ranked in the top 100 by ANTIGENpro.

**Table 3:** *E. ca*. hypothetical protein antigenicity and immunoreactivity.

Protein (Ecaj_ tag no.)	Mean ELISA OD^a^	ANTIGENpro rank	Antigenicity score	*E. ch*. ortholog/ANTIGENpro rank/Immunoreactive^b^
0919	2.29	106	0.841	1147/2/−
0126	2.13	1	0.962	0187/1/−
0717	1.85	150	0.804	*
0636	1.52	187	0.759	0377/95/−
0073	1.51	61	0.887	0122/185/+
0920	1.44	5	0.947	1148/78/+
0259	1.27	21	0.928	0825/26/+
0348	1.22	178	0.767	*
0748	0.94	157	0.797	*
0676	0.87	186	0.760	0329/434/−
0922	0.73	27	0.920	*
0723	0.60	222	0.728	*
0736	0.46	140	0.814	*
0730	0.42	116	0.831	*
0342	0.39	80	0.871	*
0767	0.32	75	0.875	*

^a^Mean ELISA OD from 10 CME dog sera.

^b^(+) Immunoreactive in immunoscreening; (−) not immunoreactive in immunoscreening; (*) *E. ch.* ortholog not identified.

Previously, we have reported that several pairs of *E. ch*./*E. ca*. orthologs, including TRP32/TRP19, TRP47/TRP36, TRP75/TRP95, and TRP120/TRP140, are all major immunoreactive proteins. Although 10 *E. ca*. and 7 *E. ch*. orthologs were identified in IMG that corresponded with the new major immunoreactive proteins, none of ortholog pairs exhibited similar immunoreactivity (Tables 2 and 3). We found that three *E. ca*. and three *E. ch*. orthologs reacted with a few of CME dog and HME patient sera, respectively, but none of these orthologs reacted consistently with HME or CME sera. These findings suggest that the antibody epitopes in the new proteins are not conserved among corresponding ortholog pairs from *E. ch*. and *E. ca*., unlike the linear antibody epitopes defined in orthologs we have previously reported^4^.

### Bioinformatic analysis of *E. ch*. and *E. ca*. immunoreactive proteins

We determined that among the 15 *E. ch*. and 16 *E. ca*. new immunoreactive proteins, 10 (67%, *E. ch*.) and 13 (81%, *E. ca*.) were small (≤250 amino acids) (Tables 4 and 5). Notably, tandem repeats were found in three *E. ch*. proteins (Ech_0700, 0252, and 0531) and 1 *E. ca*. protein (Ecaj_0126), similar to other well-known ehrlichial immunoreactive TRPs. A comprehensive bioinformatic analysis of these proteins was performed using multiple online prediction tools. By TMHMM 2.0 server (http://www.cbs.dtu.dk/services/TMHMM/), nine (60%) *E. ch*. and seven (44%) *E. ca*. proteins were predicted to contain at least one transmembrane helix. However, using SignalP 5.0 (http://www.cbs.dtu.dk/services/SignalP), a standard secretory signal peptide, which is transported by the Sec translocon and cleaved by signal peptidase I, was identified in only one protein (0846). Moreover, SecretomeP 2.0 (http://www.cbs.dtu.dk/services/SecretomeP) predicted six *E. ch*. and five *E. ca*. proteins to be secreted by a non-classical (i.e., not signal peptide directed) mechanism. Since type I and type IV secretion systems (T1SS and T4SS) are present in *E. ch*. and *E. ca*., we examined these proteins as possible T1 and T4 substrates. Sequence analysis did not identify a consensus type IV secretory motif R-X(7)-R-X-R-X-R in any of the proteins^33^. Two *E. ca* proteins (0126 and 0259) were predicted to be type IV substrates by S4TE 2.0, a new algorithm that predicts type IV effector proteins^34^; however, none of the *E. ch*. proteins were predicted to be type IV substrates. In contrast, statistical analysis of the last 50 C-terminal residues of these proteins identified a putative type I secretion signal (LDAVTSIF-enriched; KHPMWC-poor) described previously^35^, suggesting that the majority of these proteins are type I secreted substrates. The predicted type IV substrate Ecaj_0259 showed the smallest difference between the residue occurrences of LDAVTSIF (36%) and KHPMWC (24%) in the last 50 C-terminal amino acids. In addition, we further examined these proteins using the recently reported PREFFECTOR server (http://draco.cs.wpi.edu/preffector), which identifies all effectors regardless of the secretion system using a feature-based statistical framework^36^. PREFFECTOR prediction identified 10 (66%) *E. ch*. and 10 (63%) *E. ca*. proteins as effectors (probability threshold = 0.8). This analysis supports the conclusion that many of these proteins are small type I secreted effectors, of which 52% contain a transmembrane domain (Tables 4 and 5). Further experiments are required to experimentally validate them as T1SS substrates.

**Table 4:** Predicted features of new *E. ch*. immunodominant hypothetical proteins.

Protein (Ech_ tag no.)	Amino acids/mass (kDa)	Tandem repeats	Transmembrane domains (TMHMM)	Secretion (SecretomeP)	T4S (S4TE)	Effector (PREFFECTOR)
1053	193/22	−	+	−	−	+
0578	185/21	−	−	−	−	−
0846	171/19	−	−	+^a^	−	+
0745	118/13	−	−	+	−	+
0700	192/20	+	−	+	−	+
0607	322/38	−	−	−	−	+
0535	186/21	−	−	+	−	+
0716	367/41	−	+	−	−	−
0252	364/40	+	+	−	−	−
0722	190/21	−	+	+	−	+
0991	710/81	−	+	−	−	+
0240	158/18	−	+	−	−	−
0531	175/20	+	+	+	−	+
0715	551/61	−	+	−	−	−
0181	103/12	−	+	+	−	+

^a^Signal peptide predicted by SignalP.

**Table 5:** Predicted features of new *E. ca*. immunodominant hypothetical proteins.

Protein (Ecaj_ tag no.)	Amino acids/mass (kDa)	Tandem repeats	Transmembrane domains (TMHMM)	Secretion (SecretomeP)	T4S (S4TE)	Effector (PREFFECTOR)
0919	120/14	−	−	+	−	+
0126	671/78	+	−	+	+	+
0717	226/25	−	+	−	−	+
0636	98/11	−	−	−	−	−
0073	92/10	−	−	+	−	−
0920	182/20	−	−	+	−	+
0259	368/41	−	−	+	+	+
0348	535/59	−	+	−	−	−
0748	121/13	−	+	−	−	−
0676	229/26	−	−	−	−	+
0922	133/15	−	−	−	−	+
0723	248/28	−	+	−	−	−
0736	188/21	−	+	−	−	+
0730	165/18	−	−	−	−	−
0342	217/24	−	+	−	−	+
0767	91/10	−	+	−	−	+

### *E. ch*. and *E. ca*. immunoreactive protein antibody epitopes

**Fig. 4:**
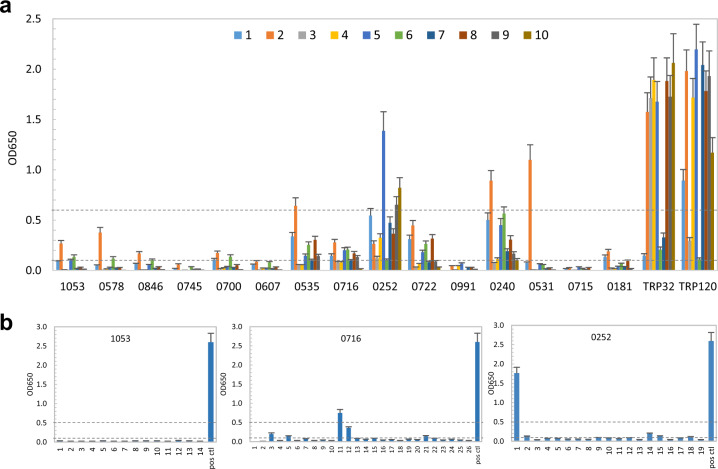
Conformation-dependent immunoreactivity of 15 recombinant *E. ch*. hypothetical proteins. **a** Immunoreactivity comparison of the denatured IVTT-expressed hypothetical proteins and TRPs by ELISA using a panel of 10 sera from HME patients. **b** Immunoreactivity of overlapping synthetic peptides spanning three immunoreactive proteins, as determined by ELISA with an HME patient serum. Positive control, TRP120. OD values represent the mean optical density reading from three wells (±standard deviations) after background subtraction. A sample OD of ≥0.1 was considered positive and ≥0.5 a strong positive after subtracting negative control (a**:** IVTT reaction with empty plasmid template and b: a negative peptide) reading.

The number of major immunoreactive *E. ch*. proteins identified by immunoblot is small and well-defined^4^. Thus, to understand how these new immunoreactive proteins are not apparent by immunoblot, we considered the possibility of conformation-dependent antibody epitopes. To examine this question, we compared the immunoreactivity of native proteins (IVTT products) with that of denatured proteins (IVTT products treated by urea) by ELISA with the same panel of sera from 10 HME patients (Fig. 4a). After denaturation, four *E. ch*. immunoreactive proteins (Ech_0745, 0607, 0991, and 0715) did not react with any patient serum; five proteins (Ech_1053, 0578, 0846, 0700, and 0181) reacted weakly with 1–3 patient sera; five proteins (Ech_0535, 0716, 0252, 0722, and 0240) reacted with most patient sera but at a substantially lower level compared to native IVTT proteins. One protein (0531) reacted strongly with a single patient serum but did not react with the other nine sera. However, the immunoreactivity of major immunoreactive TRP32 and TRP120 was not affected by denaturation, consistent with our previous reports demonstrating that TRPs contain major linear epitopes (Fig. 4a)^4^. These results indicate that these new *E. ch*. immunoreactive proteins are defined by conformation-dependent antibody epitopes.

We have previously used synthetic peptides to map linear epitopes in *E. ch*. TRPs^5,6,9^. Thus, we used this approach to further determine if new *E. ch*. immunoreactive proteins contain significant linear epitopes. Overlapping polypeptides (20–25 amino acids; 6 amino acid overlap) were synthesized to cover the sequence of 13 *E. ch*. immunoreactive proteins (Table 2; except Ech_0991 and 0715). The HME patient serum used in our initial screening was used to probe all peptides by ELISA (Fig. 4b). Several peptides from Ech_0716 (peptides 3, 5, 11, 12, and 21) and 0252 (peptides 1, 14, and 15) reacted with the HME serum. Overlapping peptides representing the remaining 11 proteins, such as Ech_1053, did not react with the HME patient serum, supporting the conclusion that a majority of these *E. ch*. immunoreactive proteins do not contain major linear epitopes, a finding consistent with our ELISA using native and denatured IVTT products (Figs 2 and 4a).

**Fig. 5:**
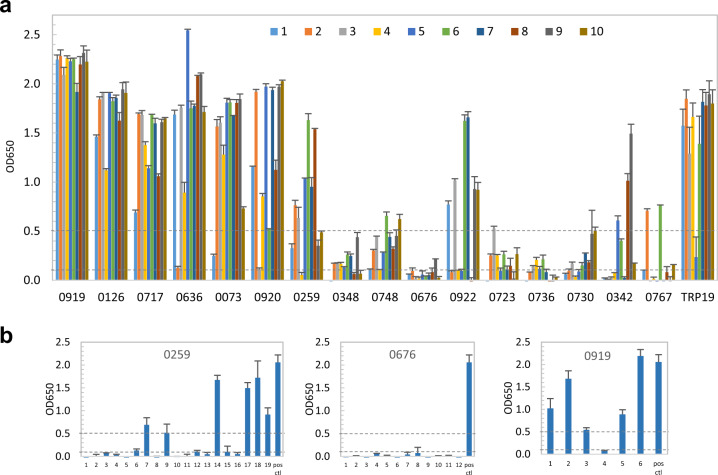
Conformation-dependent immunoreactivity of 16 *E. ca*. hypothetical proteins. **a** Immunoreactivity of the IVTT-expressed denatured *E. ca*. hypothetical proteins compared with TRP19 by ELISA. The IVTT proteins reacted with convalescent sera from 10 CME dogs. **b** Immunoreactivity of overlapping synthetic peptides spanning 3 *E. ca*. immunoreactive proteins, as determined by ELISA with a CME dog serum. Positive control, TRP19. ELISA OD values represent the mean optical density reading from three wells (±standard deviations) after background subtraction. A sample OD of ≥0.1 was considered positive and ≥0.5 a strong positive after subtracting negative control (a: IVTT reaction with empty plasmid template and b: a negative peptide) reading.

In order to examine epitope conformation-dependence of new *E. ca*. immunoreactive proteins, we compared the immunoreactivity of native proteins and denatured IVTT products by ELISA with 10 CME dog sera (Fig. 5a). After denaturation, seven *E. ca*. proteins, including Ecaj_0348, 0748, 0676, 0723, 0736, 0730, and 0767 did not react with most dog sera or reacted with sera at a substantially lower level compared to native IVTT proteins; however, the immunoreactivity of other nine *E. ca*. proteins was not reduced substantially, similar to well-defined *E. ca*. major immunoreactive protein TRP19 (Fig. 5a). Thus, our results indicate that 7 of 16 new *E. ca*. immunoreactive proteins have conformation-dependent antibody epitopes.

Additionally, we investigated conformational dependence using overlapping synthetic peptides to identify linear epitopes in three *E. ca*. proteins (Fig. 5b). By ELISA, some peptides of Ecaj_0259 (peptides 7, 9, 14, 17, 18, and 19) and 0919 (peptide 1, 2, 3, 5, and 6) reacted strongly with the CME dog serum, suggesting the presence of major linear epitopes in these two proteins. None of Ecaj_0676 peptides reacted with the dog serum, suggesting the absence of linear epitopes. These results support the conclusion that some new *E. ca*. immunoreactive proteins contain major linear epitopes while some others contain conformational epitopes (Figs 3 and 5a).

We also investigated conformational dependence in some new *E. ch*. and *E. ca*. immunoreactive proteins by dot immunoblot (Supplementary Fig. 2). The immunoreactivity of native proteins was compared with that of denatured proteins using an HME or CME serum. After denaturation, Ech_0745 did not react with the HME serum and 0535 and 0716 reacted weakly with the serum, whereas 0252 still reacted strongly with the serum but at a lower level compared to native proteins. These results are consistent with our ELISA data in Fig. 4a and support the conclusion that these *E. ch*. immunoreactive proteins are defined by conformation-dependent antibody epitopes. After denaturation, Ecaj_0919 still reacted strongly with the CME serum but Ecaj_0636, 0073, and 0676 did not react with the serum. The results of 0636 and 0073 are not consistent with our ELISA data in Fig. 5a, demonstrating that these two proteins contain conformational epitopes that refold in solution and recover the immunoreactivity after denaturing. Therefore, we conclude that the majority of new *E. ca*. immunoreactive proteins contain conformational epitopes.

In order to further confirm the conformational dependence, *E. ch*. and *E. ca*. immunoreactive proteins with linear or conformational antibody epitopes were expressed in an *E. coli* cell-based expression system and the recombinant proteins purified under native conditions and immunoreactivity examined by Western immunoblot and ELISA (Supplementary Fig. 3). After SDS–PAGE and Western immunoblot, Ech_0252 reacted strongly with antibodies in HME patient serum demonstrating the presence of a linear antibody epitope, whereas Ech_0607 was not immunoreactive due to denaturation of the conformational epitope. Similarly, by Western immunoblot Ecaj_0919 reacted strongly with antibodies in CME dog serum demonstrating the presence of a linear antibody epitope, but Ecaj_0073 was not immunoreactive. We then investigated the immunoreactivity of these proteins by ELISA. Similarly, by ELISA, the native Ech_0252 and 0607 and denatured Ech_0252 proteins reacted with antibodies in HME patient serum, while denatured Ech_0607 was not immunoreactive. Native and denatured Ecaj_0919 and 0073 proteins reacted strongly with the CME serum. Compared to Western immunoblot, denatured Ech_0073 in ELISA (in solution) demonstrated refolding and restoration of conformation and thus nearly complete restoration of immunoreactivity (Supplementary Fig. 3). These results further support the ELISA and dot immunoblot results presented in Figs 4a, 5a and S2, that concluded the majority of new *Ehrlichia* immunoreactive proteins are defined by conformation-dependent antibody epitopes.

## Discussion

The first immunoreactive *E. ch*. proteins (GroES/EL) were molecularly characterized in 1993^37^. Since that time, molecular and proteomic approaches used to identify major immunoreactive proteins of *Ehrlichia* spp. have revealed a small subset of proteins (TRPs, Anks, and OMPs) defined by immunodominant linear antibody epitopes^1^. These proteins are easily identifiable on *E. ch*. or *E. ca.-*infected mammalian cell immunoblots and have been the primary focus of immunomolecular characterization studies of these pathogens^1^. In this investigation, we used bioinformatic prediction to rank the top 250 *E. ch*. and *E. ca*. antigenic proteins and further investigated the proteins (~40%) contained in this group that have unknown function (hypothetical). In order to overcome a major barrier in identifying the immunoreactive ehrlichial proteins, we used cell-free high-throughput IVTT ORF expression. Combining these approaches, we identified many previously undiscovered immunodominant and subdominant ehrlichial proteins, most characterized by small size (<250 aa) and conformation-dependent immunoreactivity. These proteins, which have remained undefined and have the potential to be transformative in vaccine and diagnostic development and challenge our view of the immunome, represented in these obligately intracellular bacteria.

Prior to this investigation, many obstacles have impeded attempts to define the *E. ch*. and *E. ca*. immunomes including challenges in growing ehrlichiae in tick cells, lack of genome sequence information, limitations of conventional protein analysis approaches, and difficulties in studying conformational aspects of protein immunoreactivity. In this investigation, we relied on the new-generation computational and biotechnical approaches to reveal a large group of unknown immunoreactive proteins. These approaches included bioinformatic prediction (reverse vaccinology) to prioritize candidate screening, gene synthesis to overcome issues associated with low-throughput manual gene cloning, IVTT to express proteins in native conformation, particularly those that are not amenable to cell-based expression systems, and high-throughput ELISA immunoscreening to rapidly and accurately identify immunoreactive proteins. We found that *E. ch*. and *E. ca*. proteins could be expressed by IVTT, many of which could not be expressed in a cell-based expression system. With IVTT, the protein expression levels varied; however, the expression levels did not appear to impact the identification of immunoreactive proteins since many proteins with lower IVTT expression levels showed strong immunoreactivities with patient or dog sera (e.g., Ech_0745, 0607, 0578 and Ecaj_0730, 0717, 0748). This can be explained by the fact that IVTT-expressed proteins are captured by anti-His antibody-coated plates and the wells become saturated with each protein. Thus, the amount (~100 ng) of each respective protein analyzed using the ELISA is roughly equivalent, regardless of individual protein expression levels during IVTT.

We utilized a bioinformatic approach to help identify and prioritize *E. ch*. and *E. ca*. immunoreactive proteins. ANTIGENpro has been utilized successfully in previous studies to identify antigens that generate a protective humoral immune response. We used a cutoff proposed by this algorithm to develop a list of proteins with the highest antigenic scores in order to improve positive hits and increase the rate at which we could identify the most promising prospects. In our analysis, ANTIGENpro ranked known antigenic/protective proteins such as TRPs and OMPs in the top 250 list (Table 1). This finding supports previous reports that ANTIGENpro accurately predicts antigenic/protective proteins, and indicates that there was an enrichment of antigenic proteins by ANTIGENpro as has been previously reported.

The *E. ch*. and *E. ca*. genomes contain 426 (39%) and 238 (25%) genes, respectively, that encode proteins of unknown function so far. The proportion of *E. ch*. hypothetical proteins represented in the genome is nearly double that of *E. ca*. Notably, nearly half of the *E. ca*. hypothetical proteins (*n* = 98) were identified by ANTIGENpro as highly antigenic; however, only 22% (*n* = 93) of the *E. ch*. hypothetical proteins were identified as highly antigenic. The majority of known ehrlichial immunoreactive proteins, including TRPs, were initially classified as hypothetical proteins. However, our recent studies have revealed the functional aspects of TRPs, and it is now established that they are secreted effectors that interact with an array of host proteins and have various distinct functions during infection^38–41^. Hypothetical proteins have also been shown to be predominant immunoreactive proteins in other intracellular pathogens, suggesting that many antigenic and potentially protective proteins have unknown function^24,42^. Therefore, based on previous empirical data we considered hypothetical proteins to be higher priority candidates and the focus of this study. Our results of immunoscreening and antigenic protein identification support this prioritization strategy.

The *E. ch*. and *E. ca*. genomes have a large proportion of ORFs that encode small proteins (<250 aa). In fact, even if many short ORFs (<42 aa) are ignored, still nearly 10% of the *E. ch*. ORFs encode proteins <100 aa. Very few of these proteins have been investigated, and the smallest known immunoreactive protein is *E. ca* TRP19 which is 160 aa. The reasons for the large proportion of small proteins encoded by these genomes and their role in pathobiology are unclear; however, in this study, we identified many (23/31; 74%) small (<250 aa) undiscovered hypothetical immunoreactive proteins of *E. ch*. and *E. ca*. Nineteen (61%) of the immunoreactive proteins of *E. ch*. and *E. ca*. were <200 aa and three proteins were <100 aa. Conventional gel electrophoresis would not resolve such proteins well, or in many instances these proteins would be eliminated from the gel depending on the gel composition and electrophoresis conditions. These findings suggest that there has been a large group of important immunoreactive proteins that may have remained undefined in part due to difficulties in resolving such proteins with standard gel electrophoretic approaches.

All previously characterized *E. ch*. and *E. ca*. immunoreactive proteins contain major linear epitopes^4^. Yet, conformation-dependent epitopes predominated in this study, suggesting that previous approaches used to identify immunoreactive proteins were effective in revealing the immunoreactive proteins with linear antibody epitopes while leaving many immunoreactive proteins undiscovered. Identification of proteins with conformational epitopes requires special techniques such as IVTT, capable of expressing proteins in native conformation in solution, therefore, it is not surprising that many of these proteins have remained undiscovered. Using IVTT, we exposed conformation-dependent epitopes previously concealed, revealing a large group of new antigenic proteins. To our knowledge, such a large abundance of proteins with conformational epitopes has never been reported previously in other pathogens and indicates that the *Ehrlichia* immunomes have a predominance of epitopes with conformation-dependence.

Conformational antibody epitopes have been experimentally identified in human pathogens, mostly reported in viruses, such as functional epitopes on hepatitis E virions/capsids, the orf virus major envelope protein B2L and dengue virus envelope E glycoprotein^43–45^. A few conformation-dependent epitopes have also been described in bacterial proteins, such as *E. ch*. TRP32, *Yersinia pseudotuberculosis* OmpF porin, and *Campylobacter jejuni* membrane protein Cj1621^6,42,46^. These proteins have been demonstrated to play an important role in pathogen infection and eliciting host antibody response. Both linear and conformational antibody epitopes are essential in stimulating immunity; however, it has been estimated that more than 90% of B-cell epitopes are conformational, since <10% of antibodies raised against intact proteins react with peptide fragments derived from the parent protein^47^. The frequency of conformation-dependent epitopes in *Ehrlichia* spp. revealed in this investigation demonstrates the importance of such epitopes in generating an immune response and further highlights the need to fully identify and define the immunodeterminants for effective vaccines to be developed.

*Ehrlichia* spp. infect arthropod and mammalian hosts, and this host infection dynamic may have also contributed to the difficulties in uncovering these immunoreactive proteins. Prior to this study, antigens that had been discovered are known to be highly expressed in mammalian cells^18^. Previously, most investigations have relied on *Ehrlichia*-infected human/canine cells instead of tick cell cultures for antigen discovery; however, it has been demonstrated that differential expression of *Ehrlichia* antigenic proteins occurs in arthropod vs. mammalian hosts^18–20^. We also previously reported upregulated gene expression of a large number of *Ehrlichia* hypothetical proteins in tick cells compared to human cells, including Ech_0535, 0722 and 0531, and we demonstrated differential protein expression of TRPs in human vs. tick cells^18^. Others have demonstrated divergent protein immunoreactivity in tick vs. human cell-cultivated *E. ch*., illuminating the differences in *E. ch*. proteomes from distinct host cell environments^19^. Therefore, it is likely that many of these newly identified *Ehrlichia* antigens are expressed in tick cells, not in mammalian cells, and thus have escaped identification and characterization. A previous study using one-dimensional electrophoresis identified several immunoreactive hypothetical proteins expressed in mammalian and/or tick cells^19^; however, although these proteins were classified as antigenic in our investigation, we did not identify them as immunoreactive in this study. Differences observed between the previous study and the current investigation could be related to the approach of excising proteins from a gel for mass spectrometry that may include comigrating proteins rather than the direct gene expression approach used herein. In addition, previous studies have used sera from needle-inoculated mice to identify immunoreactive *E. ch*. proteins. This study used convalescent sera from tick-transmitted HME patients and CME dogs to identify immunoreactive proteins. Moreover, we used methods capable of identifying proteins that contain conformational epitopes, which were not used previously. Thus, using this approach we have identified many undiscovered proteins that may be useful as immunodiagnostics for detection of early antibodies that are elicited by tick-expressed ehrlichial proteins or for developing transmission-blocking or infection-blocking subunit vaccines.

Most of the immunoreactive proteins previously identified from *E. ch*. and *E. ca*. consist of ortholog pairs, such as TRP32/TRP19, TRP47/TRP36, TRP75/TRP95, TRP120/TRP140, and Ank200/Ank200, all of which contain major linear epitopes, suggesting that ehrlichiae have similar orthologous immunomes^48^. However, in contrast to previously established similarity between immunoreactive orthologs, ortholog pairs in this study did not react similarly and consistently with antibodies in sera from *E. ch*.-infected patients and *E. ca*.-infected dogs. Moreover, many of the new *E. ch*./*E. ca*. immunoreactive proteins do not have corresponding orthologs. Since most of these proteins exhibited conformational epitopes, this difference highlights a divergence in antibody recognition that is fundamentally different from previously defined linear epitopes in major immunoreactive proteins of *Ehrlichia*. These results further demonstrated that *E. ch*. and *E. ca*. have vastly different conformational immunomes that are not shared between the species, a finding in contrast with previously defined linear epitope containing proteins. This newly recognized diversity in immunomes has potential importance in development of effective vaccines and provides new insight into the feasibility of developing cross protective vaccines. In addition, we compared the sequences of the top *E. ch*. and *E. ca*. immunoreactive proteins (Tables 2 and 3) among different sequenced strains, including nine *E. ch*. genome sequences (US strains) and two *E. ca*. genome sequences (US Jake and China HZ-1) that are available. We determined that these immunoreactive proteins are present in all *E. ch*. or *E. ca*. strains and are highly conserved.

Tandem repeats were identified in four immunoreactive proteins (three in *E. ch*. and one in *E. ca*.) and this observation further highlights the importance of ehrlichial TRPs as targets of the host immune response. In addition, although many of the new immunoreactive proteins of *E. ch*. are predicted to be secreted, they also contain transmembrane domains which are considered a feature of membrane proteins. The significance of transmembrane domains in secreted effector proteins remains to be determined, but this is an interesting and unique feature that, to our knowledge, has not been previously described.

In this study, the majority of new immunoreactive proteins were predicted to be secreted, but only two *E. ch*. proteins (Ecaj_0126 and 0259) were predicted to be T4S substrates by S4TE. *Ehrlichia* spp. have type I and type IV secretion systems, which are both common among Gram-negative bacteria. Substantial emphasis has been placed on identification of T4 effectors in many different bacteria including *Ehrlichia*; however, we have previously demonstrated the importance of type I secretion system in *Ehrlichia* spp. and have identified numerous T1SS substrates, including TRPs and Ank200 that are also major immunoreactive proteins^49^. TRPs interact with multiple host proteins associated with conserved cell biological processes, including cell signaling, transcriptional regulation, vesicle trafficking, cytoskeleton organization, and apoptosis^48,50^. Similarly, the majority of new immunoreactive proteins are predicted to be effectors, suggesting that these proteins may be involved in many different interactions with the host cell during infection. The fact that these *Ehrlichia* T1SS substrates are immunoreactive proteins suggests that such effectors are predominantly targeted by the host immune response, which may be related to the importance of neutralizing their functional properties to limit infection. Further studies are needed to confirm whether these immunoreactive *E. ch*. proteins are indeed type I-secreted effectors and to determine their role in ehrlichial pathobiology.

The new *Ehrlichia* proteins identified in this study significantly expand the number of identified major immunoreactive proteins and highlights the potential importance of conformational antibody epitopes in immunity and differential expression of ehrlichial proteins in mammalian and tick hosts. We have identified major immunoreactive proteins that have immunoreactivity that rivals TRPs. These immunoreactive proteins provide additional options for developing highly sensitive methods for diagnosis of HME and CME or markers to distinguish vaccinated from non-vaccinated subjects. More importantly, these immunoreactive proteins may be important in transcending a long-standing barrier that has impeded subunit vaccine development for HME and CME.

## Methods

### ANTIGENpro prediction

The *E. ch*. (Arkansas strain) and *E. ca*. (Jake strain) ORFeomes were analyzed by ANTIGENpro (http://scratch.proteomics.ics.uci.edu/), a sequence-based and alignment-free predictor of protein antigenicity^29^. The predictions are made by a two-stage architecture based on multiple representations of the primary sequence and five machine learning algorithms. A final score (0–1) defines the antigenic probability, with a higher score correlating with increased antigenicity. A prediction threshold of ~0.7, which provides maximum sensitivity and specificity, was used to identify the most highly antigenic proteins in *E. ch*. and *E. ca.*^29^.

### Gene synthesis

*E. ch*. or *E. ca*. genes in this study are available by locus tag identification in the integrated microbial genomes (IMG) (http://img.jgi.doe.gov/)^51^. The ORFs were obtained by either PCR amplification or chemical gene synthesis. For PCR, *E. ch*. (Arkansas) or *E. ca*. (Jake) was propagated and purified for genomic DNA preparation^18,52,53^. Oligonucleotide primers for the amplification of the gene fragments were designed manually or by PrimerSelect (Lasergene 13, DNASTAR, Madison, WI) according to the sequences and synthesized (Integrated DNA Technologies, Coralville, Iowa). PCR was performed with PCR HotMaster Mix (Eppendorf, Westbury, NY) using *E. ch*. or *E. ca*. genomic DNA as the template. The thermal cycling profile was: 95 °C for 3 min, 30 cycles of 94 °C for 30 s, annealing temperature (1 °C less than the lowest primer *T*_m_) for 30 s, and 72 °C for the appropriate extension time (1 min/1000 base pairs) followed by a 72 °C extension for 10 min and a 4 °C hold. Chemical synthesis of *E. ch* and *E. ca*. genes was performed by GenScript (Piscataway, NJ) or Biomatik (Wilmington, DE).

### HME and CME antisera

HME patient sera were kind gifts from the Centers for Disease Control and Prevention (Atlanta, GA), Vanderbilt University (Nashville, TN), Washington University, and St. Louis Children’s Hospital (St. Louis, MO). CME dog sera were obtained from naturally infected dogs from the United States and Colombia. Convalescent sera and anti-IgG secondary antibodies were used in this study to examine the immunoreactivity to avoid the possibility of polyreactive antibodies (IgM) which have been previously described^54^.

### IVTT and dot immunoblot

In vitro expression of ehrlichial proteins was performed using the S30 T7 high-yield protein expression system (Promega, Madison, WI), an *E. coli* extract-based cell-free protein synthesis system which can produce a high level of recombinant protein in vitro. Briefly, the ehrlichial ORFs were cloned in pIVEX-2.3d or pET-14b vector containing T7 promoter/terminator and a 6×His-tag sequence. The recombinant plasmid was mixed with a *E. coli* extract and a reaction premix that contain all necessary components for transcription and translation, such as T7 RNA polymerase and ribosomal machinery, followed by the incubation at 37 °C for 2 h. The IVTT expression of ehrlichial ORFs was confirmed by dot immunoblotting IVTT-expressed products (1 μl each) on nitrocellulose. The membrane was incubated with horseradish peroxidase (HRP)-labeled His-tag mouse antibody (1:500; GenScript) in Tris-buffered saline (TBS) with 3% nonfat dry milk and 0.1% Tween 20 for 1 h at room temperature. The membrane was washed three times with TBS, and the protein was visualized after adding TMB 1-component substrate (Kirkegaard & Perry Laboratories, Gaithersburg, MD) and incubated for 15 min. To examine the immunoreactivity by dot immunoblot, IVTT-expressed ehrlichial proteins were purified by MagneHis protein purification system (Promega) according to the instructions from the manufacturer and reacted with an HME or CME antiserum.

### ELISA immunoscreening

A His-tag antigen-capture ELISA was used to screen *E. ch*. and *E. ca* His-tag IVTT-expressed proteins for immunoreactivity. Briefly, His-tag antibody plates (GenScript) were blocked with 100 μl of StartingBlock^TM^ (PBS) blocking buffer (Thermo Fisher) with 2% nonfat milk for 20 min and washed twice with 200 μl of phosphate-buffered saline containing 0.05% (v/v) Tween 20 (PBST). The plates were coated with 50 µl of diluted (1:50) IVTT reaction mixture in dilution buffer (blocking buffer with 2% nonfat milk and 0.05% Tween 20) and incubated overnight at 4 °C. The plates were washed five times with PBST using an Immunowash 1575 microplate washer (Bio-Rad, Hercules, CA). HME patient or CME dog sera diluted (1:200) in dilution buffer were added to each well (50 μl) and incubated for 1 h. ELISA plates were washed again, and 50 μl of alkaline phosphatase-labeled rabbit anti-human IgG (H + L) secondary antibody (1:5000; Abcam, Cambridge, MA) in dilution buffer was added, and incubated for 1 h. After final washes, 100 μl of BluePhos substrate (Kirkegaard & Perry) was added and the plates were incubated in the dark for 30 min. Optical density (OD) was determined using a VersaMax microplate reader (Molecular Devices, Sunnyvale, CA) at *A*_650_ and data analyzed by Softmax Pro 7 (Molecular Devices). Immunoreactivity of denatured IVTT-expressed proteins was examined similarly except the dilution buffer contained 4 M urea, and the mixture was incubated for 10 min at 99 °C before coating the ELISA plates. ELISA OD values represent the mean OD reading from three wells (±standard deviations) after background subtraction. Since negative controls generally had raw readings of <0.08 OD, a sample OD of ≥0.1 was considered positive and ≥0.5 a strong positive after subtracting negative control reading.

### Peptide ELISA

Peptide ELISAs to identify linear antibody epitopes in *E. ch*. and *E. ca*. immunoreactive proteins were performed using overlapping peptides (six amino acids overlapped) commercially synthesized by Bio-Synthesis (Lewisville, TX) or Biomatik^5^. All peptides were supplied as a lyophilized powder and resuspended in molecular biology grade water (1 mg/ml).

### Indirect fluorescent-antibody assay (IFA)

The antibody titers of sera from HME patients and CME dogs were determined by IFA. Antigen slides were prepared from THP-1 cells infected with *E. ch*. (Arkansas) or DH82 cells infected with *E. ca*. (Jake). Sera were diluted two-fold in PBS, starting at 1:100. Fifteen microliters of diluted serum was added to each well, and then the wells were incubated for 30 min. Slides were washed, and a fluorescein isothiocyanate (FITC)-labeled rabbit anti-human or dog IgG (H + L) secondary antibody (Kirkegaard & Perry Laboratories) diluted 1:100 was added, and the mixture was incubated for 30 min. Slides were viewed with a BX61 epifluorescence microscope (Olympus, Japan).

### Cell-based recombinant protein expression and Western immunoblot

Ehrlichial genes were amplified and cloned into the pBAD/Thio-TOPO expression vector (Invitrogen, Carlsbad, CA) and expressed in *E. coli* TOP10 cells (Invitrogen)^5^. Recombinant protein was purified under native conditions using HisSelect columns (Sigma, St. Louis, MO) according to the instructions from the manufacturer. To examine the immunoreactivity of recombinant ehrlichial proteins, Western immunoblot was performed with HME or CME antiserum^15^. All blots or gels were derived from the same experiment and that they were processed in parallel.

### Reporting summary

Further information on research design is available in the Nature Research Reporting Summary linked to this article.

## Supplementary information

Supplementary Information

Reporting Summary

## Data Availability

The data supporting the findings of the study are available from the corresponding author upon reasonable request.
