# Supplementary material for: Ehrlichia chaffeensis and E. canis hypothetical protein immunoanalysis reveals small secreted immunodominant proteins and conformation-dependent antibody epitopes
Source: NPJ Vaccines. 2020 Sep 11;5:85. doi: 10.1038/s41541-020-00231-1 (PMC7486380; doi:10.1038/s41541-020-00231-1)
Supplement: Supplementary file 1 — Supplementary Information [file 41541_2020_231_MOESM1_ESM.pdf]

Supplementary Table 1. Highly antigenic *E. ch.* hypothetical proteins (n=93) by ANTIGENpro (score ≥ 0.695).

No.	Ech_ tag no.	Antigenicity score	Mean ELISA OD	Size (AA)	*E.ca.* ortholog (Ecaj_ tag no.)
1	0187	0.969	0.06	563	0126
2	1147	0.964	0.05	126	*
3	0247	0.958	0.05	302	*
4	0261	0.956	1.08	264	0762
5	0255	0.950	0.89	338	0764
6	0253	0.950	0.01	189	*
7	0865	0.949	0.04	302	0229
8	1152	0.949	0.04	185	0923
9	0722	0.945	0.41	190	*
10	0246	0.944	0.03	275	*
11	0257	0.943	0.02	226	*
12	0609	0.935	0.01	301	*
13	0601	0.929	0.00	374	0434
14	0535	0.928	0.20	186	0500
15	0251	0.928	0.41	205	*
16	0576	0.924	0.04	98	0462
17	0150	0.923	0.05	672	0099
18	1037	0.920	0.02	1231	0835
19	0745	0.920	0.21	118	0324
20	0864	0.918	0.13	330	0231
21	0825	0.917	0.52	380	0259
22	0113	0.909	0.03	793	*
23	0166	0.908	0.50	285	0109
24	0862	0.907	0.00	403	0232
25	0531	0.905	0.42	175	*
26	0285	0.895	0.20	181	*
27	0744	0.889	0.06	157	0325
28	0612	0.888	0.00	208	*
29	0879	0.885	0.03	815	*
30	0147	0.885	0.47	193	0096
31	0611	0.880	0.19	229	0428
32	1036	0.880	0.04	750	0834
33	0525	0.879	0.01	666	0508
34	0252	0.875	0.61	364	*
35	0118	0.873	0.00	30	*
36	0807	0.864	0.05	334	0271
37	0348	0.862	0.12	202	0660
38	0763	0.860	0.33	165	0312
39	0106	0.858	0.00	713	0066
40	1154	0.857	0.14	135	0926

41	0120	0.857	0.00	213	*
42	0240	0.857	0.31	158	*
43	1148	0.854	0.21	142	0920
44	0243	0.853	0.02	293	*
45	0284	0.852	0.03	1016	0716
46	0115	0.851	0.00	203	*
47	0345	0.850	0.36	294	0663
48	0878	0.847	0.00	409	*
49	1021	0.845	0.02	219	0824
50	0700	0.845	0.15	192	*
51	0607	0.844	0.31	322	0434
52	0377	0.843	0.02	104	0636
53	0549	0.842	0.15	195	*
54	0614	0.839	0.54	231	0423
55	1103	0.830	0.26	223	0881
56	0846	0.828	0.48	171	0242
57	0199	0.823	0.00	213	0136
58	0108	0.819	0.01	825	0072/0071
59	0551	0.811	0.17	191	0479
60	1027	0.804	0.00	34	*
61	0663	0.802	0.14	202	0379
62	0578	0.798	0.37	185	*
63	0716	0.790	0.64	367	0347
64	0778	0.786	0.15	1132	0297
65	1013	0.785	0.06	203	0818
66	0398	0.781	0.41	121	0621
67	0991	0.779	0.70	710	0139
68	0927	0.775	0.02	34	*
69	0949	0.773	0.21	31	*
70	0259	0.773	0.36	118	*
71	0704	0.771	0.35	248	0351
72	0256	0.770	0.01	72	*
73	0181	0.769	0.15	103	0122
74	0297	0.769	0.07	272	0706
75	0388	0.768	0.71	293	*
76	0159	0.767	0.50	507	0104
77	1053	0.763	0.61	193	0846
78	0122	0.758	0.10	126	0073
79	0593	0.758	0.10	382	0445
80	0698	0.758	0.01	200	*
81	0079	0.756	0.01	134	0047
82	0986	0.752	0.16	179	0142
83	0715	0.748	0.14	551	0348

84	0279	0.747	0.01	41	*
85	0836	0.737	0.13	1201	0253
86	0281	0.716	0.39	179	*
87	0276	0.716	0.06	184	*
88	0526	0.715	0.07	495	0507
89	0478	0.704	0.29	172	0548
90	0126	0.704	0.04	334	0077
91	0866	0.703	0.06	330	0228
92	0945	0.699	0.00	1349	0174
93	0767	0.695	0.02	621	0309

* *E.ca.* ortholog not identified.

Supplementary Table 2. Highly antigenic *E. ca.* hypothetical proteins (n=98) by ANTIGENpro (score ≥ 0.710).

No.	Ecaj_ tag no.	Antigenicity score	Mean ELISA OD	Size (AA)	*E.ch.* ortholog (Ech_ tag no.)
1	0126	0.962	2.23	671	0187
2	0341	0.951	0.16	190	*
3	0920	0.947	0.90	182	1148
4	0715	0.945	0.06	1918	*
5	0099	0.945	0.02	630	0150
6	0923	0.942	0.27	184	1152
7	0063	0.940	0.10	705	*
8	0762	0.940	0.01	353	0261
9	0503	0.939	0.19	110	*
10	0069	0.938	0.04	823	*
11	0838	0.932	0.06	1510	1038
12	0431	0.929	0.03	436	*
13	0259	0.928	1.75	368	0825
14	0346	0.926	0.01	518	*
15	0071	0.925	0.51	641	0121
16	0500	0.925	0.00	185	0535
17	0716	0.925	0.15	1601	0284
18	0324	0.922	0.00	117	0745
19	0922	0.920	1.56	133	*
20	0067	0.919	0.00	695	*
21	0764	0.919	0.01	191	0255
22	0660	0.917	0.05	197	0348
23	0139	0.915	0.07	794	0991
24	0220	0.915	0.22	461	*
25	0230	0.915	0.04	486	*
26	0772	0.914	0.10	163	*
27	0924	0.912	0.06	144	1152
28	0066	0.912	0.02	889	0106
29	0835	0.911	0.08	1267	1037
30	0325	0.906	0.00	157	0744
31	0068	0.905	0.01	616	*
32	0881	0.904	0.00	305	1103
33	0462	0.903	0.00	93	0576
34	0228	0.899	0.09	345	0866
35	0072	0.899	0.03	916	0121
36	0312	0.897	0.00	188	0763

37	0345	0.897	0.05	631	*
38	0356	0.891	0.00	85	*
39	0824	0.890	0.24	242	1021
40	0073	0.887	1.70	92	0122
41	0379	0.886	0.10	182	0663
42	0528	0.885	0.00	199	0500
43	0743	0.885	0.00	84	*
44	0271	0.884	1.17	329	0807
45	0434	0.881	0.00	223	0601
46	0834	0.876	0.08	782	1036
47	0767	0.875	0.79	91	*
48	0065	0.871	0.08	595	*
49	0342	0.871	0.51	217	*
50	0232	0.868	0.16	412	0862
51	0062	0.867	0.04	957	*
52	0096	0.864	1.29	194	0147
53	0620	0.861	0.00	97	0399
54	0429	0.861	0.00	251	*
55	0771	0.852	0.06	245	*
56	0231	0.849	0.09	328	0864
57	0508	0.843	0.18	621	0525
58	0919	0.841	2.35	120	1147
59	0343	0.841	0.14	181	*
60	0450	0.840	0.02	330	*
61	0229	0.838	0.10	354	0865
62	0309	0.837	0.06	840	0767
63	0726	0.835	0.11	214	*
64	0730	0.831	0.67	165	*
65	0741	0.830	0.19	114	*
66	0428	0.829	0.07	290	0611
67	0625	0.821	0.02	112	0391
68	0926	0.820	0.06	135	1154
69	0185	0.819	0.17	271	0929
70	0663	0.818	0.02	293	0345
71	0736	0.814	0.60	188	*
72	0445	0.812	0.07	382	0593
73	0297	0.807	0.02	1130	0778
74	0717	0.804	2.06	226	*
75	0423	0.801	0.10	257	*

76	0047	0.801	0.02	133	0079
77	0748	0.797	1.75	121	*
78	0186	0.794	0.10	285	0929
79	0381	0.788	0.05	184	0660
80	0493	0.782	0.06	239	0540
81	0239	0.778	0.14	951	*
82	0142	0.776	0.03	178	0986
83	0348	0.767	1.35	535	*
84	0430	0.761	0.07	183	*
85	0676	0.760	0.65	229	0329
86	0636	0.759	1.70	98	0377
87	0122	0.756	0.03	103	0181
88	0347	0.756	0.78	354	0716
89	0482	0.756	0.02	92	*
90	0254	0.755	0.03	269	0835
91	0739	0.748	0.02	220	*
92	0253	0.739	0.05	1206	0836
93	0174	0.729	0.03	1306	0945
94	0725	0.728	0.03	190	*
95	0723	0.728	0.46	248	*
96	0727	0.714	0.02	97	*
97	0119	0.712	0.20	881	0176
98	0198	0.710	0.12	381	0907

* *E.ch.* ortholog not identified.

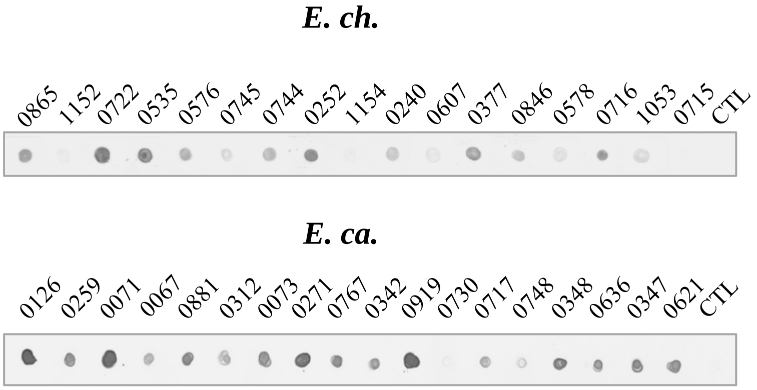

Supplementary Fig. 1 Expression of *E. ch.* and *E. ca.* hypothetical proteins by IVTT. Detection of IVTT expression of randomly selected hypothetical proteins of *E. ch.* (upper) and *E. ca.* (bottom) by dot immunoblot with anti-His-tag antibody. CTL, the negative control (IVTT reaction without plasmid template).

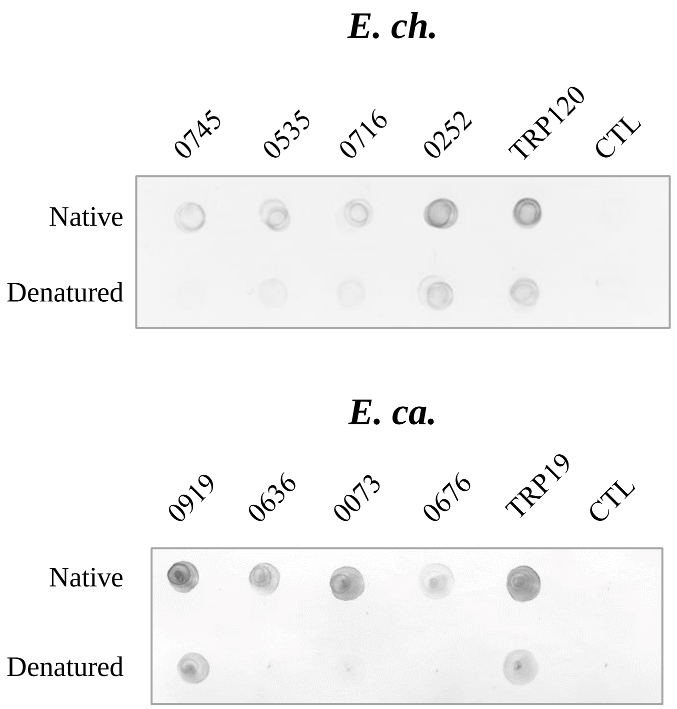

Supplementary Fig. 2 Conformation-dependent immunoreactivity of *E. ch.* and *E. ca.* hypothetical proteins. Immunoreactivity of the native and denatured hypothetical proteins and TRPs was detected by dot immunoblot with serum from an HME patient or CME dog. All proteins were IVTT-expressed and purified. CTL, the negative control (IVTT protein with empty plasmid template).

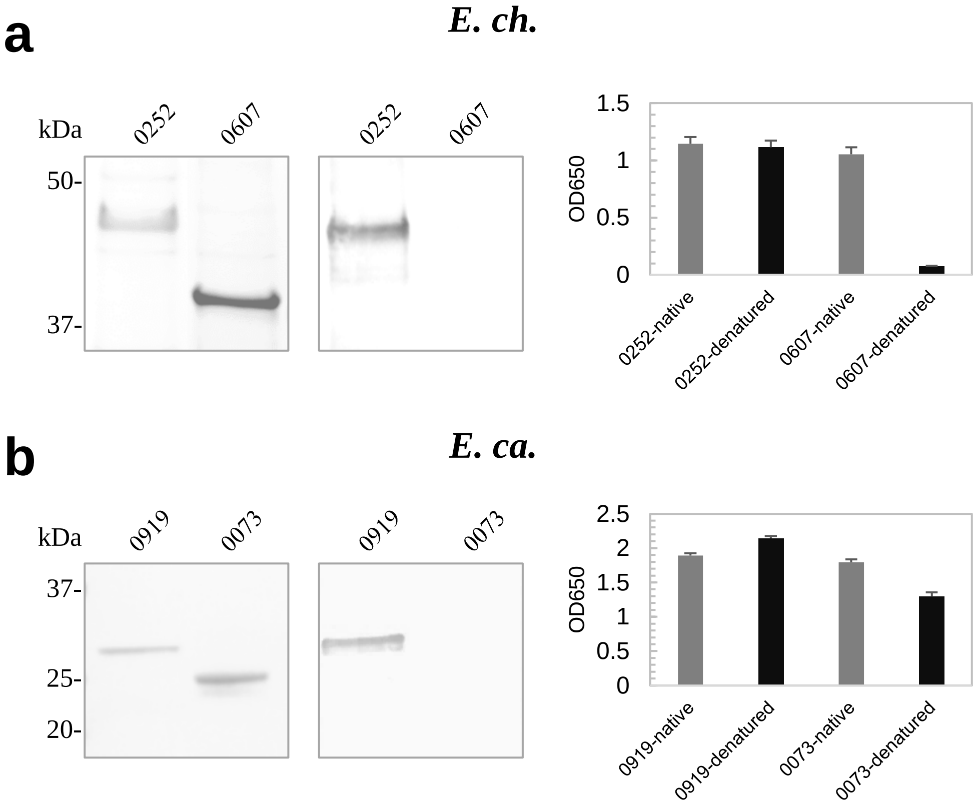

Supplementary Fig. 3 Conformation-dependent immunoreactivity of *E. coli*-expressed *E. ch.* and *E. ca.* recombinant proteins. Native and denatured recombinant *E. ch.* (**a**) and *E. ca.* (**b**) proteins were expressed in *E. coli* and purified and probed with serum from an HME patient or CME dog and detected by Western immunoblot and ELISA. Left panel, SDS-PAGE of purified recombinant proteins stained with Coomassie blue; middle panel, Western immunoblot; right panel, ELISA OD values. ELISA OD values represent the mean optical density reading from 3 wells (± standard deviations) after background subtraction.
